# Remote programming for subthalamic deep brain stimulation in Parkinson's disease

**DOI:** 10.3389/fneur.2022.1061274

**Published:** 2022-11-24

**Authors:** Si Chen, Shu-jun Xu, Wei-guo Li, Teng Chen, Chao Li, Shuo Xu, Ning Yang, Yi-ming Liu

**Affiliations:** Center for Movement Disorders, Qilu Hospital of Shandong University, Jinan, China

**Keywords:** Parkinson's disease, deep brain stimulation, subthalamic nucleus, remote programming, China, activities of daily living (ADL)

## Abstract

**Introduction:**

Deep brain stimulation (DBS) of the subthalamic nucleus (STN) is effective for the treatment of Parkinson's disease (PD). Moreover, remote programming is widely used in Mainland China. This necessitates evaluating the ability of remote programming to achieve the ideal postoperative effect. Therefore, we aimed to retrospectively evaluate the effects of different programming modes on the effectiveness of STN-DBS 12 months postoperatively in patients with PD.

**Methods:**

Clinical data were collected retrospectively, before and 12 months after surgery, in 83 patients with PD. Based on the programming modes voluntarily selected by the patients during 12 months postoperatively, they were divided into three groups, namely remote programming alone, hospital programming alone, and hospital + remote programming. We compared the programming data and the effects of different programming methods on STN-DBS-related improvements 12 months postoperatively among these groups. Furthermore, we analyzed STN-DBS-related improvements at 12 months postoperatively in 76 patients.

**Results:**

The effectiveness of STN-DBS was not influenced by the three programming modes. The postoperative Movement Disorder Society Unified Parkinson's Disease Rating Scale scores did not reveal statistically significant differences between the remote alone and hospital alone programming groups, except for motor examination. The postoperative decline in the levodopa equivalent daily dose was most apparent in the hospital programming alone group. The programming frequency of the hospital + remote programming group was considerably higher than that of the remaining groups. Seventy-six patients with PD displayed good STN-DBS surgical efficacy.

**Conclusion:**

Programming modes do not influence the short-term efficacy of STN-DBS, and remote programming can yield a satisfactory surgical effect.

## Introduction

Deep brain stimulation (DBS) of the subthalamic nucleus (STN) is reportedly effective for the treatment of Parkinson's disease (PD) (1, 2). DBS can effectively improve the off-medication motor symptoms of patients with PD, relieve their motor complications, reduce the dose of levodopa, and significantly enhance their quality of life. Currently, the STN is the most widely used target for DBS in PD (3). The effectiveness of DBS primarily depends on patient selection, accurate electrode implantation, and effective postoperative programming. Of these factors, effective programming can ensure high postoperative efficacy and patient satisfaction, besides preventing stimulation-related adverse events (4).

With advancements in DBS hardware, researchers have developed several novel programming technologies, of which remote programming is widely used in Mainland China (5, 6). Remote programming platforms enable programming physicians to perform the parameter adjustment of postoperative patients at home, thereby improving patient convenience. Since 2018, we have been performing remote programming for patients exhibiting PD using PINS products (PINS Medical, Ltd., Beijing, China) at the Movement Disorders Center of the Qilu Hospital of Shandong University, Shandong, China. Following DBS implantation, patients can voluntarily select to undergo hospital programming, remote programming, or a combination of both. The remote programming platform is provided by PINS and operated by associating the remote physician client and mobile patient client through the Internet. The Bluetooth connection between the mobile patient client and the implantable pulse generator (IPG) can be used to facilitate audio/video communication, patient parameter adjustments, and electrode impedance checks (7). In addition, programming data from previous remote programming can be queried through the database of this platform.

Remote programming not only reduces the travel and financial burden of the patients but also offers advantages, such as highlighting the need for urgent MRI examinations or the treatment for disease changes as well as facilitating patient management despite travel restrictions owing to diseases or various causes. These advantages have been particularly relevant in recent years considering the coronavirus disease 2019 (COVID-19) pandemic. Nevertheless, programming is one of the most important factors influencing the postoperative efficacy of DBS; thus, the ability of remote programming to achieve the ideal postoperative effect is of great significance. Therefore, we aimed to analyze retrospectively the effects of different programming modes on the efficacy of STN-DBS in patients with PD 12 months postoperatively. Moreover, we aimed to provide a preliminary report of the advantages and disadvantages of remote programming in terms of its clinical application.

## Methods

### Patient enrollment

This study included 83 patients with PD undergoing bilateral STN-DBS in the Department of Neurosurgery of Qilu Hospital of Shandong University between June 2018 and December 2019. The inclusion criteria were as follows: (1) PD diagnosis by movement disorder experts according to the Movement Disorder Society (MDS) clinical diagnostic criteria (8); (2) meeting the surgical indications for DBS and excluding surgical contraindications (9). All 83 patients selected DBS equipment with a remote programming function (IPG: G102R; electrode model: L301, PINS). This study was approved by the Ethics Committee of the Qilu Hospital of Shandong University [approval number: KYLL-2017(KS) 270], and written informed consent was obtained from all participants.

### Data collection and grouping

Clinical data were retrospectively collected before and 12 months after surgery of all patients with PD. The data included pre-surgical general data, MDS-Unified Parkinson's Disease Rating Scale (UPDRS) scores, levodopa equivalent daily dose (LEDD), and postoperative programming data. The MDS-UPDRS consists of four parts. The first and second parts assess non-motor aspects (nM-EDL) and motor aspects of the experiences of daily living (M-EDL), respectively. Contrarily, parts III and IV involve motor examination and evaluations of motor complications (dyskinesia, motor fluctuations and “OFF” dystonia), respectively (10). The programming data included the programming frequency during 12 months, stimulation settings for the voltage, pulse width, and frequency, and the proportion of interleaved stimulation during 12 months post-surgery. Both high- (HFS, 130–185 Hz) and low-frequency stimulation (LFS, 60–100 Hz) were used in programming to control different symptoms. Therefore, we compared the results obtained with LFS and HFS in the three groups. Moreover, we measured the home-hospital distances of the three groups.

The IPG was turned on according to the recommended process 2 to 4 weeks post-surgery (11), and the remote programming clients were installed during face-to-face assessments in the hospital. The patients voluntarily selected their preferred programming mode and provided their programming-related requirements. Two professional programming physicians performed all on-site and remote programming procedures free of charge every Tuesday.

Based on the programming modes voluntarily selected during 12 months postoperatively, the patients were divided into three groups as follows: remote programming alone group, hospital programming alone group, and hospital + remote programming group. We compared the effects of different programming methods on the improvement in these patients 12 months following DBS among the three groups. Furthermore, we compared the postoperative programming data among the three groups. We analyzed improvements in all patients 12 months following STN-DBS; however, we could not obtain the MDS-UPDRS motor examination scores following surgery from the patients who received remote programming alone owing to the unavailability of physical examination data obtained through video communication.

### Statistical methods

Measurements are expressed as mean ± standard deviation and percentile (P25–P75) for normally distributed and non-normally distributed variables, respectively. Intergroup comparisons were performed using the analysis of variance (or the non-parametric test. Intergroup comparisons of categorical data were performed by the chi-squared test or Fisher's exact probability test. In postoperative intergroup comparisons, we analyzed the effects of different programming modes on the efficacy of DBS using a linear mixed model. Pairwise comparisons for the variables displaying statistically significant differences were performed using the Student-Newman-Keuls method. Statistical analyses were performed using SPSS 22.0 (IBM SPSS Inc., Chicago, USA).

## Results

### Preoperative clinical characteristics

Of the 83 patients undergoing bilateral STN-DBS, the hospital programming alone group, remote programming alone group, and hospital + remote programming group comprised 25, 23, and 35 patients, respectively. Table 1 summarizes a comparison of preoperative data.

**Table 1:** Preoperative clinical characteristics.

	**Hospital** **programming** **group (*n* = 25)**	**Remote** **programming** **group (*n* = 23)**	**Hospital + remote** **programming** **group (*n* = 35)**	***P*** **-value**
Males/Females	19/6	12/11	18/17	0.119
Age at surgery (years)	62.28 ± 8.28	60.04 ± 5.73	61.26 ± 8.84	0.622
Age at PD onset (years)	51.28 ± 9.61	49.09 ± 7.09	53.29 ± 9.05	0.205
Disease duration (years)	11.04 ± 7.55	10.96 ± 4.64	8.46 ± 3.46	0.104
**MDS-UPDRS (scores)**
nM-EDL	13.60 ± 5.42	13.43 ± 5.54	12.74 ± 6.30	0.832
M-EDL	22.96 ± 6.03	23.57 ± 6.86	22.51 ± 8.43	0.868
Motor examination (off medication)	55.12 ± 15.15	54.48 ± 21.29	47.06 ± 18.03	0.165
Motor examination (on medication)	26.84 ± 12.20	24.39 ± 14.16	22.37 ± 13.79	0.450
Dyskinesias	0.0 (0.00–2.00)	0.00 (0.00–2.00)	1.00 (0.00–3.00)	0.858
Motor fluctuations	6.20 ± 2.71	6.91 ± 1.46	6.74 ± 1.96	0.481
“OFF” dystonia	0.00 (0.00–1.00)	0.00 (0.00–2.00)	0.00 (0.00–0.00)^a^	0.008
LEDD (mg/d)	942.90 ± 315.28	1021.14 ± 444.51	867.32 ± 334.39	0.289
Distance from the hospital (km)	133.68 ± 124.32^a^	255+129.04	165.37 ± 100.96^a^	0.002

MDS-UPDRS, Movement Disorder Society-Unified Parkinson's Disease Rating Scale; LEDD, levodopa equivalent daily dose; nM-EDL, non-motor aspects of experiences of daily living; M-EDL, motor aspects of experiences of daily living; and PD, Parkinson's disease.

a In comparison with the remote programming group, P < 0.05.

Before surgery, the three groups did not reveal significant differences in the age, sex, the age of onset, the course of the disease, the age of surgery, motor symptoms, LEDD, and scores of the four parts of MDS-UPDRS (except “OFF” dystonia). These findings indicated no significant intergroup differences in the general data, the non-motor symptoms of PD, the activities of daily living, and motor examination findings before surgery. In addition, an assessment of home-hospital distances suggested that the home-hospital distance of the patients who selected remote programming alone was significantly longer than in those of the remaining two groups.

### Effects of different programming modes

Owing to the characteristics of the remote programming mode, we could not obtain motor examination data for patients who underwent remote programming alone. Moreover, of the 23 patients who selected receive remote programming alone; postoperative 12-month follow-up data were unavailable for seven patients (Table 2).

**Table 2:** A comparison of the postoperative clinical characteristics among the three groups undergoing bilateral STN–DBS.

	**Hospital** **programming** **group (*n* = 25)**	**Remote** **programming** **group (*n* = 16)**	**Hospital + remote** **programming** **group (*n* = 35)**	***P*** **-value**
**MDS-UPDRS (scores)**
nM-EDL	9.82 ± 0.88	10.29 ± 1.09	11.66 ± 0.74	0.251
M-EDL	12.94 ± 1.30	13.00 ± 1.62	15.96 ± 1.10	0.142
Motor examination (off medication)	24.90 ± 2.02	–	29.81 ± 1.70	0.071
Dyskinesias	0.48 ± 0.27	1.10 ± 0.34	0.81 ± 0.23	0.338
Motor fluctuations	3.78 ± 0.49	4.93 ± 0.61	4.19 ± 0.41	0.341
“OFF” dystonia	0.36 ± 0.13	0.25 ± 0.17	0.20 ± 0.11	0.643
LEDD (mg/d)	401.54 ± 37.55	462.10 ± 46.99	570.86 ± 31.83^a^	0.003
LEDD reduction (%)	53.40 ± 4.46	45.48 ± 7.52	33.34 ± 3.55^a^	0.006

LEDD, levodopa equivalent daily dose; nM–EDL, non-motor aspects of experiences of daily living; M-EDL, motor aspects of experiences of daily living; and STN–DBS, subthalamic nucleus deep brain stimulation.

a In comparison with the hospital programming group, P < 0.05.

The clinical data obtained 12 months post-surgery demonstrated that the different programming modes did not affect the primary effectiveness of STN-DBS, including outcomes related to non-motor symptoms, the activities of daily living, and motor complications. Moreover, the motor examinations did not reveal statistically significant differences between the hospital programming alone group and the hospital + remote programming group. The postoperative MDS-UPDRS scores did not demonstrate statistically significant differences between the two programming alone modes, except for motor examination. Notably, the postoperative decline in LEDD in the hospital programming alone group was more apparent than that in the combined group, with the two groups displaying a statistically significant difference.

### Stimulation settings

We analyzed the stimulation settings, including the voltage, pulse width, and frequency in the three groups 12 months postoperatively (Table 3). The voltage did not display significant differences among the three groups. The pulse width of the remote programming alone group was narrower than that of the hospital + remote programming group. The frequency was higher in the hospital programming alone group than that in the remaining two groups. No significant differences were observed in the proportion of LFS or the frequency of patients using LFS among the three groups. Of the patients using HFS, the frequency in the hospital programming alone group was significantly higher than that in the remaining two groups. There were no statistically significant differences in the proportion of interleaved stimulation among the three groups. Moreover, the mean programming frequency within 12 months of surgery was 3.13, 2.52, and 5.09 times in the remote programming alone group, hospital programming alone group, and hospital + remote programming group, respectively. The programming frequency within 12 months in the hospital + remote programming group was significantly higher than those in the remaining two groups.

**Table 3:** Stimulation settings.

	**Hospital** **programming** **group (*n* = 25)**	**Remote** **programming** **group (*n* = 23)**	**Hospital + remote** **programming** **group (*n* = 35)**	***P*** **-value**
Frequency of programming (*n*)	2.52 ± 0.22	3.13 ± 0.42	5.09 ± 0.390^a^^b^	<0.001
Voltage (V)	2.34 ± 0.40	2.43 ± 0.36	2.44 ± 0.48	0.341
Pulse width (μs)	66.18 ± 9.33	63.75 ± 5.90	67.87 ± 9.83^a^	0.024
Frequency (Hz)	147.73 ± 31.69	133.13 ± 27.89^b^	133.48 ± 25.41^b^	0.006
LFS/HFS	3/22	3/20	6/29	0.818
LFS (Hz)	92.50 ± 4.63	90.00 ± 0.0	93.85 ± 8.25	0.269
HFS (Hz)	156.9 ± 23.97	142.5 ± 21.15^b^	139.0 ± 22.26^b^	<0.001
Interleaved stimulation, *n* (%)	4 (16.00)	8 (34.78)	11 (31.43)	0.283

LFS, Low-frequency stimulation; HFS, high–frequency stimulation.

a In comparison with the remote programming group, P < 0.05.

b In comparison with the hospital programming group, P < 0.05.

### Improvement at 12 months following STN-DBS

Clinical data at 12 months following surgery were obtained from 76 patients. The LEDD reductions and scores for non-motor symptoms, the activities of daily living, motor examination, dyskinesia, motor fluctuations, and “OFF” dystonia were significantly different from those obtained before the surgery (Table 4).

**Table 4:** Improvements in 76 patients with PD 12 months post–surgery.

	**n**	**Pre–surgical**	**12-months post-surgery**	***P*** **-value**
**MDS-UPDRS (scores)**
nM-EDL	76	13.00 ± 5.82	10.76 ± 5.03	0.001
M-EDL	76	22.074 ± 7.28	14.34 ± 7.25	<0.001
Motor examination (off medication)	60	50.42 ± 17.23	27.77 ± 11.38	<0.001
Dyskinesias	76	0.00 (0.00–2.00)	0.00 (0.00–1.75)	0.011
Motor fluctuations	76	6.57 ± 2.18	4.21 ± 2.56	<0.001
“OFF” dystonia	76	0.00 (0.00–0.75)	0.00 (0.00–0.00)	0.042
LEDD (mg/d)	76	912.24 ± 326.92	492.27 ± 232.39	<0.001

LEDD, levodopa equivalent daily dose; nM-EDL, non-motor aspects of experiences of daily living; M-EDL, motor aspects of experiences of daily living; and PD, Parkinson's disease.

## Discussion

Remote programming is a novel technology for DBS that has emerged in recent years. Currently, remote programming technology is used in PINS and SceneRay products in Mainland China (12, 13), and its application has extended to vagus nerve stimulation for the treatment of epilepsy (14). Furthermore, other centers in China have reported on the application of remote programming and confirmed its safety and effectiveness (7, 15). Ma et al. followed up patients receiving remote programming for an average of 27 months, thus confirming remote programming is timely and effective in DBS, besides offering economic benefits for the patients (7). During the COVID-19 pandemic, two Chinese scholars reported on the use of remote programming to meet the programming needs of patients exhibiting PD; they achieved high satisfaction rates (16, 17). A study on telemedicine in patients with movement disorders during the COVID-19 outbreak in China (17) revealed an increase in the number of patients receiving DBS telemedicine in February 2020 and March 2020; the majority of patients (89%) reported satisfactory results.

Considering the longer home-hospital distances of patients who selected only remote programming, inconvenient travel may be a reason for this selection. The number of patients and programming frequency of the hospital + remote programming group were higher than those in the remaining two groups. This finding may be related to greater programing requirements or more programing mode options for these patients.

The three programming modes did not affect the primary efficacy of STN-DBS in such patients. The outcomes of motor symptoms are typically used to determine the effect of STN-DBS (1, 3). However, we did not perform motor examination of the remote programming alone group because the patients did not visit the hospital for follow-up after turning on the IPG. Therefore, we predominantly used the second part of the MDS-UPDRS to compare the postoperative motor aspects of daily living in 16 patients of the remote programming alone group, which partially reflected their motor ability. There were no differences in the postoperative MDS-UPDRS scores, in addition to the motor examination between the two programming alone modes. Thus, remote programming can achieve results similar to that of hospital programming. Furthermore, this phenomenon could explain the continuous implementation of remote programming. Xu et al. (16) assessed the postoperative motor symptoms based on the data and video recorded by patients' caregivers, which are an expedient strategy; however, it is difficult to obtain accurate results. In addition, incomplete follow-up data were obtained in the remote programming alone group. In the present study, 7 of 23 patients in the remote programming alone group did not gain regular follow-up data. Therefore, the patients who selected only remote management could weaken the management of patients following DBS.

In terms of postoperative LEDD reduction, LEDD reduction in the hospital programming alone group was more apparent than that in the remaining two groups; it demonstrated a significant difference from that in the hospital + remote programming group. Programming and drug adjustments play complementary roles in the management of postoperative patients with PD. Usually, LEDD can be reduced by 30 to 50% following STN-DBS (11). The LEDD in the remote programming alone group, hospital programming alone group, and hospital + remote programming group reduced by 45.48, 53.40, and 33.34%, respectively, which was consistent with previous data. During hospital programming, patients can obtain neurologists' suggestions on drug reduction in the hospital. However, considering the safety of unwritten medication adjustments and the limitations of prescription drugs, the programming physicians adjusted the medications less in remote programming than that in hospital programming. This phenomenon is a disadvantage of remote programming and has not been addressed in previous studies. Considering these disadvantages, we propose that the Neuromodulation Center should cooperate with the local hospitals to conduct drug adjustment and postoperative data follow-up.

In our analysis of postoperative programming settings, the groups did not demonstrate significant differences in the voltage; however, they displayed differences in the pulse width and frequency. Voltage is the most important programming factor for improving motor symptoms following STN-DBS. Furthermore, an increase in the pulse width can improve the symptoms; nonetheless, the improvement was not as apparent as that achieved with changes in the voltage (18). The relevance of frequency programming following DBS in patients with PD is a controversial issue. HFS can improve their motor symptoms, whereas LFS can improve their gait and axial symptoms (19). However, an exact definition of low frequency remains unavailable. In this study, LFS was defined by frequencies <100 Hz. The use of LFS among the three groups did not reveal significant differences; however, the frequency in the hospital programming group (156.9 ± 23.97 Hz) was significantly higher than that in the remaining two groups (142.5 ± 21.15 and 139.0 ± 22.26 Hz) with the use of HFS. Moreover, these differences are clinically insignificant with the use of HFS. Professor Moro demonstrated that increasing the frequency between 130 and 185 Hz gradually improved the motor symptoms; however, the improvement was statistically insignificant (18). Moreover, the use of interleaved stimulation presented no significant differences among the three groups. Recently, PINS has begun providing variable frequency stimulation to improve the freezing of gait and axial symptoms (20, 21). Nonetheless, we did not use this technology, which may be attributed to its report in few cases and a small sample size. Moreover, no specific guidelines have been proposed for its usage.

STN-DBS can significantly improve the activities of daily living and motor symptoms of patients with PD, besides significantly reducing the LEDD (1, 22, 23). Furthermore, we analyzed the postoperative improvements in 73 patients exhibiting PD with complete data, thus revealing good results for LEDD reduction, non-motor symptoms, the activities of daily living, motor symptoms, dyskinesia, motor fluctuations, and “OFF” dystonia, consistent with the findings of previous reports.

We should acknowledge the several limitations of the present study. First, the hospital + remote programming group had more patients than the other two groups and seven patients were lost to follow-up in the remote programming alone group. The second limitation was related to the short follow-up period. The efficacy of DBS gradually declines in patients with PD because of the exacerbation of axial symptoms and disease progression (22). Following the onset of axial symptoms with poor DBS efficacy (24), the patients may request multiple programming adjustments or changes to the programming mode. In cases displaying axial symptoms or other troublesome symptoms in programming, we recommend multiple attempts and observations to identify the optimal stimulation settings (25). In such cases, the patients are recommended to select hospital programming. Therefore, remote programming should be performed as a supplement for hospital programming, which cannot replace hospital programming. In addition, the programming physicians at our center conducted the initial and follow-up DBS programming procedures according to the expert consensus on programming following DBS for PD and previous recommendations (11, 25). However, the personal experience levels of the programming physicians also affect the programming frequency and the choice of programming mode.

In conclusion, the programming modes did not affect the short-term efficacy of STN-DBS. Moreover, remote programming yielded a satisfactory surgical effect. Through cooperation with local hospitals, remote programming can be not only convenient and effective but also achieve better postoperative management. Thus, remote programming technology may provide a novel development direction for DBS in the future.

## Data availability statement

The original contributions presented in the study are included in the article/supplementary material, further inquiries can be directed to the corresponding author/s.

## Ethics statement

The studies involving human participants were reviewed and approved by Ethics Committee of Qilu Hospital of Shandong University. The patients/participants provided their written informed consent to participate in this study.

## Author contributions

S-jX, Y-mL, and SC contributed to the conception and design of the study. S-jX, W-gL, TC, CL, SX, and NY performed the surgery. SC and Y-mL performed the programming-related tasks. SC conducted the statistical analysis and drafted the manuscript. All authors have approved the submitted version.

## Funding

This work was supported by the National Science Fund of China (Grant Numbers 82171245 and 81771373). The funding agencies had no role in the study design, the collection, analysis, or interpretation of data, the writing of the report, or the decision to submit the article for publication.

## Conflict of interest

The authors declare that the research was conducted in the absence of any commercial or financial relationships that could be construed as a potential conflict of interest.

## Publisher's note

All claims expressed in this article are solely those of the authors and do not necessarily represent those of their affiliated organizations, or those of the publisher, the editors and the reviewers. Any product that may be evaluated in this article, or claim that may be made by its manufacturer, is not guaranteed or endorsed by the publisher.
